# Anemia status, hemoglobin concentration and outcome after acute stroke: a cohort study

**DOI:** 10.1186/1471-2377-10-22

**Published:** 2010-04-09

**Authors:** David Tanne, Noa Molshatzki, Oleg Merzeliak, Rakefet Tsabari, Maya Toashi, Yvonne Schwammenthal

**Affiliations:** 1 Stroke Center, Department of Neurology, the Chaim Sheba medical center, Tel- Hashomer, and the Sackler Faculty of Medicine, Tel Aviv University, Tel Aviv. Israel

## Abstract

**Background:**

In the setting of an acute stroke, anemia has the potential to worsen brain ischemia, however, the relationship between the entire range of hemoglobin to long-term outcome is not well understood.

**Methods:**

We examined the association between World Health Organization-defined admission anemia status (hemoglobin<13 in males, <12 g/dl in women) and hemoglobin concentration and 1-year outcome among 859 consecutive patients with acute stroke (ischemic or intracerebral hemorrhage).

**Results:**

The mean baseline hemoglobin concentration was 13.8 ± 1.7 g/dl (range 8.1 - 18.7). WHO-defined anemia was present in 19% of patients among both women and men. After adjustment for differences in baseline characteristics, patients with admission anemia had an adjusted OR for all-cause death at 1-month of 1.90 (95% CI, 1.05 to 3.43) and at 1-year of 1.72 (95% CI, 1.00 to 2.93) and for the combined end-point of disability, nursing facility care or death of 2.09 (95% CI, 1.13 to 3.84) and 1.83 (95% CI, 1.02 to 3.27) respectively. The relationship between hemoglobin quartiles and all-cause death revealed a non-linear association with increased risk at extremes of both low and high concentrations. In logistic regression models developed to estimate the linear and quadratic relation between hemoglobin and outcomes of interest, each unit increment in hemoglobin squared was associated with increased adjusted odds of all-cause death [at 1-month 1.06 (1.01 to 1.12; p = 0.03); at 1-year 1.09 (1.04 to 1.15; p < 0.01)], confirming that extremes of both low and high levels of hemoglobin were associated with increased mortality.

**Conclusions:**

WHO-defined anemia was common in both men and women among patients with acute stroke and predicted poor outcome. Moreover, the association between admission hemoglobin and mortality was not linear; risk for death increased at both extremes of hemoglobin.

## Background

Low hemoglobin, or anemia is a common condition among older adults, with prevalence increasing with age. Anemia is associated with increased mortality [1-4], disability, and poorer physical performance [5] regardless of the underlying cause of the low hemoglobin. In patients with acute coronary syndromes as well as in patients with angina, anemia is an independent indicator of short and long-term mortality [6-8]. At the other end of the range of hemoglobin, patients with an acute coronary syndrome or congestive heart failure with high hemoglobin values also exhibit excess mortality, suggesting a non-linear reverse J-shaped relationship [8,9]. Among community dwelling elderly, findings are inconsistent but some have observed a similar relationship with mortality [1,3] as well as with a lower level of cognitive function [10].

The relationship between the entire range of hemoglobin and outcome after stroke is not well understood. Given the relevance of hemoglobin to oxygen carrying capacity, inflammatory processes, oxidative stress, as well as to blood viscocity and cerebral blood flow, we hypothesized that extremes of both low and high hemoglobin are associated also with poor outcome in the setting of an acute stroke. Our aims were, therefore, to evaluate in a large cohort of patients hospitalized for an acute stroke: a. the predictive value of admission anemia status; b. the association between the entire range of hemoglobin concentration and stroke outcome.

## Methods

A prospective cohort study was conducted on 883 consecutive patients hospitalized due to acute stroke in a large medical center with a catchment area of about 500,000 people from March 2001 to June 2002. Baseline hemoglobin was missing in 24 patients, thus the final study cohort included 859 patients. Patients were evaluated systematically for risk factors, stroke severity, type and subtype. Risk factors were assessed from medical records and self-report. Severity of stroke was assessed using the National Institutes of Health Stroke Scale (NIHSS) [11]. Intracerebral hemorrhage and ischemic stroke were differentiated by the results of the baseline head CT scan. Ischemic stroke etiology was determined by the TOAST classification, a system for categorization of subtypes of ischemic stroke mainly based on etiology that has been developed for the Trial of Org 10172 in Acute Stroke Treatment [12]. A clinical evaluation and personal interview was performed after 1-months and a phone follow-up interview after 1-year, after obtaining informed consent. The study was approved by the local Institutional Review Board.

### Laboratory methods

Hemoglobin was measured with the Beckman Coulter MAXM/HMX analyzer (Beckman Coulter, Miami, FL) using the manufacturer's reagents and methods. The first hemoglobin measurement performed at hospital admission served as the index hemoglobin. Anemia was defined by the World Health Organization (WHO) criteria as a hemoglobin concentration <12 g/dl in women and <13 g/dl in men. Chronic kidney disease was defined according to the National Kidney Foundation and American Heart Association with a glomerular filtration rate < 60 mL/min/1.73 m^2^, estimated by the 4 variables modification of diet in renal disease equation [13], from baseline serum creatinine measured at a central laboratory using the Jaffe assay.

### Outcomes of interest

Outcomes were assessed during the first year after the stroke. During hospitalization and at 1-month follow-up, data were collected by clinical evaluation and personal interviews with patients and/or proxies. One-year after the stroke, phone follow-up interviews were conducted by professional interviewers from the Israeli Center for Disease Control blinded to in-hospital data. Functional outcome were assessed using the Barthel Index [14] with a score <75 regarded as dependent in daily activities. Mortality data were derived from the Israeli Population Registry. The main outcome of interest was all-cause death. In addition, the combined end-point of disability, nursing facility care or all-cause death was assessed.

### Statistical analyses

Anemia was analyzed according to the WHO criteria and hemoglobin concentrations by gender-specific quartiles: First, <12.3 in women, <13.3 in men; second, 12.3-13.1 in women, 13.3-14.2 in men; third, 13.2-14.1 in women, 14.3-15.2 in men; forth, ≥ 14.2 in women, ≥ 15.3 g/dl in men. Unadjusted event rates for end-points of interest by anemia status and gender-specific quartiles were compared using χ^2 ^test. To examine the relationship between anemia status and outcomes of interest, logistic regression models were conducted. The shape of the relationship between hemoglobin and event rates for end-points of interest was examined by plotting gender-specific hemoglobin quartiles against the percentage of each end-point. The plots showed a quadratic relationship. Then, logistic regression models were developed to estimate the linear and quadratic relation between hemoglobin and outcomes of interest. The variable hemoglobin was centered on its respective mean. All models were adjusted for age, gender, stroke type, stroke severity (NIHSS), prior disability, chronic kidney disease, other cardiac disease and malignancy. To determine whether the relationship of hemoglobin to mortality was influenced by gender, we repeated the adjusted regression models by adding the interaction of each hemoglobin term with gender. To visualize the relation between hemoglobin and all-cause death as demonstrated in the final logistic regression model the Loess method was used. The Loess method is a locally weighted scatterplot smoothing that helps determining the shape of the function that best summarizes the scatter plot between 2 continuous variables [15]. The method requires the input of a "smoothing parameter," which is the fraction of the data that are used around each point. An algorithm for choosing an optimal value for the smoothing parameter according to objective criteria, described by Hurvich and Simonoff [16], was used. For 1 year survival Cox proportional hazard model was used to calculate adjusted HR. All analyses were performed with SAS statistical software version 8.2 (SAS, Inc, Cary, NC).

## Results

The characteristics of the 859 study patients are shown in Table 1. The mean baseline hemoglobin concentration was 13.8 ± 1.7 g/dl (range 8.1 - 18.7). Age was negatively correlated with hemoglobin (r = -0.18, p < 0.01), and hemoglobin concentration was lower among women (13.2 ± 1.6) than among men (14.2 ± 1.6 g/dl; p < 0.01). Hemoglobin was not associated with stroke type or stroke severity, but was lower among those with prior disability, chronic kidney disease, cardiac disease (congestive heart failure or atrial fibrillation or valvular heard disease) and malignancy than those without. WHO-defined anemia was present in 19% of patients among both women and men.

**Table 1:** Baseline Characteristics of Study Cohort.

	N = 859
Age (mean ± SD) years	70.6 ± 12.5
range	29-100
Women	362(42.2%)
Type of event	
Ischemic stroke	726 (84.6%)
Intracerebral hemorrhage	132 (15.4%)
Stroke severity (NIH stroke scale)	
<5	426 (50.4%)
6 - 10	173 (20.5%)
>11	247 (29.2%)
Prior stroke	232 (27.0%)
Prior disability	265 (31.7%)
Hypertension	605 (70.5%)
Dyslipidemia	299 (34.9%)
Diabetes mellitus	281 (32.8%)
Chronic kidney disease	305 (38.0%)
Coronary heart disease (angina pectoris and/or myocardial infarction)	268 (31.2%)
Other cardiac disease (congestive heart failure, atrial fibrillation or valvular heart disease)	226 (26.3%)
Peripheral artery disease	71(8.3%)
Malignancy	78 (9.1%)

Continuous variables are expressed as mean ± SD. Categorical variables as number (%)

Mortality data, the main outcome of interest, were available for all patients. At 1-month after the event 728 patients (85%) were alive, and 586 (80%) of them had a follow-up evaluation. At 1-year, 670 patients (78%) were alive, and 501 (75%) of them had a phone follow-up interview. The age, gender and stroke severity of patients with available follow-up data at 1-months and those with available data at 1-year were comparable to the entire study cohort.

### Anemia status and clinical outcome

Clinical outcomes by anemia status are presented in Table 2. Patients with anemia exhibited poorer outcomes at both 30-day and 1-year. Similar associations were observed in men versus women, in younger versus older patients, in patients with ischemic stroke versus intracerebral hemorrhage, with no evidence for an interaction. Because of the imbalances in some baseline characteristics, multivariable logistic regression was used to evaluate the relationship between anemia status with outcomes of interest after adjusting for potential confounders (Table 3). Patients with admission WHO-defined anemia had an adjusted OR for all-cause death after 1-month of 1.90 (95% CI, 1.05 to 3.43) and after 1-year of 1.72 (95% CI, 1.00 to 2.93) and for the combined end-point of disability, nursing facility care or death of 2.09 (95% CI, 1.13 to 3.84) and 1.83 (95% CI, 1.02 to 3.27) respectively. Adjusted HR for 1-year survival was 1.48 (95% CI, 1.04 to 2.10)

**Table 2:** Clinical Outcomes by Anemia Status.

	Anemia status		
End Point	Yes n = 163	No n = 695	P-value
***At 1-Month***			
All-cause death (%)	39 (23.9%)	92 (13.2%)	<0.01
Disability, nursing facility or death (%)	89 (65.9%)	262 (44.7%)	<0.01
***At 1-Year***			
All-cause death (%)	54 (33.1%)	135 (19.4%)	<0.01
Disability, nursing facility or death (%)	82 (66.1%)	225 (46.3%)	<0.01

**Table 3:** Adjusted Odds-Ratios (95% Confidence Intervals) for Outcomes of Interest by Anemia Status.

	Anemia status	
End Point	Yes	No
***At 1-Month***		
All-cause death (%)	1.90 (1.05 - 3.43)	1.0
Disability, nursing facility or death (%)	2.09 (1.13 - 3.84)	1.0
***At 1-Year***		
All-cause death (%)	1.72 (1.00 - 2.93)	1.0
Disability, nursing facility or death (%)	1.83 (1.02 - 3.27)	1.0

Models were adjusted for age, gender, stroke type, stroke severity (NIH stroke scale), prior disability, chronic kidney disease, other cardiac disease and malignancy.

### Entire range of hemoglobin concentrations and clinical outcome

**Figure 1:**
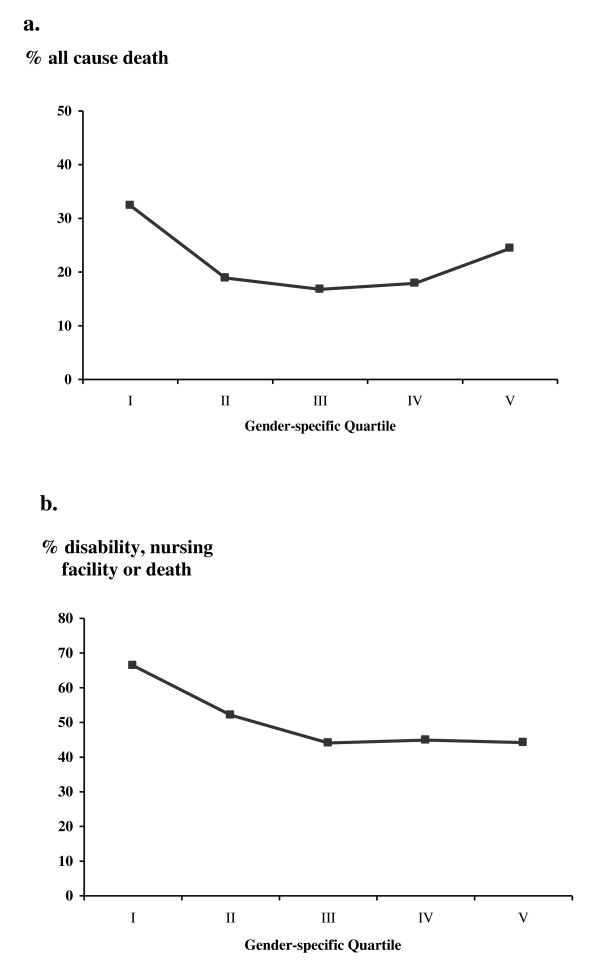
**Outcome after 1-year by gender-specific quintiles of admission hemoglobin**. a. all-cause death rate; b. disability, nursing facility care or death.

The relationships between gender-specific quartiles of hemoglobin and outcomes of interest at 1-year are depicted in Figure 1. Patients with hemoglobin in the bottom quartile exhibited the highest rates of poor outcome. Those in the middle quartiles exhibited the lowest rates, while for patients with hemoglobin in the top quartile, rates of all-cause death increased again. For the combined end-point of disability, nursing facility care or death, patients in the bottom gender-specific quartile exhibited the highest rates while rates were lower across the other quartiles.

**Figure 2:**
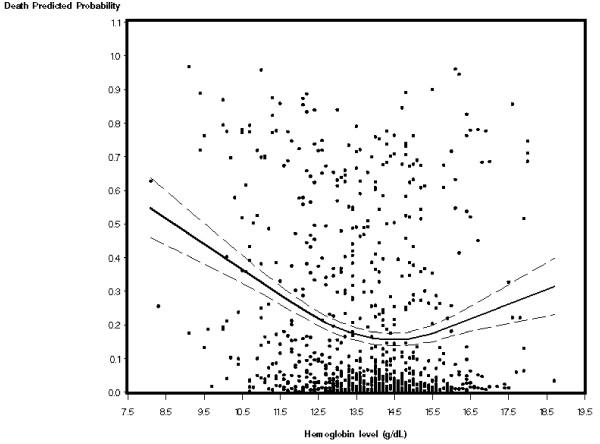
**Loess curve (with 95% confidence intervals) of hemoglobin concentrations versus the estimated probability all-cause death after 1-year**.

Based on our study hypothesis and since the shape of the relationship between hemoglobin and all-cause death showed a quadratic relationship, logistic regression models were developed to estimate the linear and quadratic relation between hemoglobin and outcomes of interest (Table 4). Each unit increment in hemoglobin squared was associated with increased adjusted odds of all-cause death [after 1-month 1.06 (1.01 to 1.12; p = 0.03); after 1-year 1.09 (1.04 to 1.15; p < 0.01)], confirming that extremes of both low and high levels of hemoglobin were associated with increased mortality. Adjusted HR for 1-year survival was 1.05 (95% CI, 1.02 to 1.09, p < 0.01). As gender may influence the relationship of hemoglobin to mortality, the model was repeated testing for interaction of each hemoglobin term with gender, but no interactions were identified. To visualize the relation between hemoglobin and the probability to all-cause death as demonstrated in the final logistic regression model the Loess method was used. As depicted in Figure 2, the curve reveals a non-linear association with increased rates at both extremes of hemoglobin concentration.

**Table 4:** Adjusted Odds-Ratios (95% Confidence Intervals) for Outcomes of Interest by Hemoglobin Squared

End Point	Hemoglobin × Hemoglobin	P-value
***At 1-Month***		
All-cause death	1.06 (1.005, 1.12)	0.03
Disability, nursing facility or death	1.07 (1.001, 1.14)	0.05
***At 1-Year***		
All-cause death	1.09 (1.04, 1.15)	<0.01
Disability, nursing facility or death	1.06 (0.998, 1.13)	0.06

Models were adjusted for age, gender, stroke type, stroke severity (NIH stroke scale), prior disability, chronic kidney disease, other cardiac disease and malignancy.

## Discussion

In this cohort of patients with acute stroke followed for 1-year, WHO-defined anemia was common in both men and women, and a significant association was found between anemia and risk for poor outcome. This relationship did not differ between men and women or between patients with intracerebral hemorrhage versus ischemic stroke type. Moreover, the association between admission hemoglobin and mortality was not linear; risk for death increased at both extremes of hemoglobin. Hemoglobin concentrations were inversely correlated with age and were lower among those with prior disability, chronic kidney disease, cardiac disease and malignancy, yet the non-linear association with mortality remained after adjusting for these potential confounders as well as for stroke type and severity.

WHO-defined anemia was present in about a fifth of patients in our cohort, and was a robust predictor of mortality over 1-year. This finding corroborates a small prior study of 250 ischemic stroke patients, observing that anemia was a negative prognostic factor [17]. Anemia is a particularly common condition among older adults, with prevalence increasing with age. Anemia in the elderly is associated with increased mortality, poorer health-related quality of life, disability, and poorer physical performance [1-5]. Among patients with an acute coronary syndrome, rates of anemia are similar to those observed in our cohort [8,18], and anemia status is associated with poor outcome [7,8,19,20].

High hemoglobin concentrations have been associated with carotid atherosclerosis and may represent a risk factor for ischemic stroke [21,22], but data on their association with outcome after acute stroke are particularly scarce and inconclusive. Sacco et al. found in the population-based L'Aquila registry that high hematocrit might represent an independent predictor of early mortality in women with ischemic stroke but not in men [23], In a group of patients with an acute ischemic stroke studied with multimodal magnetic resonance imaging, higher hematocrit had a significant independent association with reduced reperfusion and greater infarct size, suggesting that elevated levels may be a potential physiologic determinant of reduced penumbral salvage [24]. A pilot study on the association between baseline hematocrit at the time of the ischemic stroke and the discharge destination showed that midrange concentrations were associated with a better discharge outcome but were not related to mortality rate [25].

The increased mortality observed in our cohort of stroke patients at both extremes of hemoglobin is in agreement with prior observations in cardiac patients and with some but not all reports among community dwelling elderly [1,3]. It remains unclear whether the impact of hemoglobin on mortality after stroke operates through the same or different causal pathways at higher vs. lower values. Anemia may induce hypoxia at the most vulnerable regions when hemoglobin is reduced below a critical level, while high hemoglobin, may increase blood viscosity, affect cerebral blood flow and is associated with pulmonary disease. Moreover, chronically high values might enhance cerebral atherogenesis heading to a diseased and dysfunctional collateral bed [21].

Potential limitations of this study should be considered. First, it is an observational study and therefore causality cannot be inferred from the observed associations. The effects of unmeasured confounding variables such as nutritional status, frailty, cognitive function or complex interactions between covariates on the observed association cannot be ruled out. Second, we were unable to examine whether the relationship was due to increased blood loss, impaired red blood cell production, and/or increased red blood cell destruction (in the case of low hemoglobin levels) or increased red cell production (in the case of high hemoglobin levels). Third, the use of blood transfusions was not documented and was based on overall clinical judgment. Admission hemoglobin concentrations were, however, rarely in the range leading to blood transfusion in routine clinical practice. Finally, we did not measure erythropoietin levels in these patients. In addition to stimulating erythrocyte precursors, erythropoietin has multiple neuroprotective effects [26,27], and thus, some of our observations may be due to low or high levels of erythropoietin or other intervening factors rather than hemoglobin per se.

The influence of red blood-cell transfusion on clinical outcomes in selected patients with acute stroke and low hemoglobin has not been tested in trials nor discussed in guidelines. The potential benefits of transfusion by increasing oxygen-carrying capacity must be counterbalanced by risks of acute complications including infection, hemolytic reactions, volume overload, and further activation of adverse biological or inflammatory processes [28]. Achieving higher hemoglobin through pharmacologic treatment with erythropoietin-stimulating proteins was found to increase mortality, at least for anemia due to chronic kidney disease [29]. Hemodilution therapy was not found to improve overall survival or functional outcome in acute ischemic stroke, based on a systematic review of the hemodilution trials [30]. Future areas of investigation should evaluate the causes of increased mortality in individuals with low and high hemoglobin concentrations, and to assess whether aiming to a target range of hemoglobin concentrations may improve long-term outcome after stroke.

## Conclusions

We conclude that WHO-defined anemia is common in both men and women among patients with acute stroke and predicted poor outcome. Moreover, the association between admission hemoglobin and mortality is not linear; risk for death increased at both extremes of hemoglobin. Most importantly, our work suggests that hemoglobin levels (both low and high) may need to be considered as a potential factor in determining outcome after stroke.

## List of abbreviations

NIHSS: National Institutes of Health Stroke Scale; WHO: World Health Organization.

## Competing interests

The authors declare that they have no competing interests.

## Authors' contributions

DT conceived the study, participated in its design and drafted the manuscript. OM, RT, MT, and YS participated in data collection, interpretation and reviewed the manuscript. NM performed the statistical analysis. All authors read and approved the final manuscript.

## Pre-publication history

The pre-publication history for this paper can be accessed here:

http://www.biomedcentral.com/1471-2377/10/22/prepub
